# Healthcare resource accessibility and farmers’ health in rural China: a multidimensional perspective

**DOI:** 10.3389/fpubh.2026.1792629

**Published:** 2026-04-09

**Authors:** Shiyu Sun, Changcai Qin

**Affiliations:** School of Economics and Management, Yantai University, Yantai, China

**Keywords:** economic accessibility, farmer health, geographic accessibility, rural China, social accessibility

## Abstract

Farmers’ health capital is vital for global agriculture, yet farmers face multifaceted health risks. Using the China Family Panel Studies (CFPS) and China Statistical Yearbook data, this study employs fixed-effects, instrumental-variable, and quantile regression models to examine how geographic, economic, and social accessibility to healthcare affect farmers’ health in China. Economic accessibility exerts the largest and most consistent effect, particularly among older farmers and those in central and western regions. Geographic accessibility shows a counterintuitive negative association, revealing reverse causality where poorer health drives long-distance care-seeking. Social accessibility yields modest gains, becoming more important among healthier farmers and uniquely significant in the western region. Heterogeneity analysis shows stronger associations among farmers with lower educational attainment, especially for geographic and social accessibility. For lower-income farmers, financial protection alone may be insufficient and should be complemented by health education. By constructing a multidimensional framework and identifying subgroup-specific effects, this study provides evidence for targeted policies integrating financial protection, infrastructure investment, and community-embedded care to address heterogeneous barriers across rural China.

## 1 Introduction

Farmers’ health capital is a cornerstone of global agricultural systems and food security. However, farmers are among the most health-vulnerable populations worldwide, facing complex and multifaceted risks. Farmers’ physical health is influenced by occupational characteristics (1), work environment (2), and lifestyle (3), while their mental health is shaped by profession-specific stressors such as the uncertainty of climate change, fluctuating agricultural market prices, heavy debt burdens, and social isolation, which create conditions conducive to depression, anxiety, and substance abuse (4).

Research on farmers’ health at the regional level remains limited, particularly studies focusing on accessibility. Accessibility is crucial because it is an integral part of the built environment, and because it is modifiable through planning and public policy, it warrants attention as a potential population-level intervention. Existing studies on healthcare accessibility have primarily centered on geographic and economic dimensions, with much less attention to social accessibility and its influence on health outcomes. For example, geographic accessibility has been shown to significantly impact health outcomes, with rural populations facing considerable barriers due to long distances and poor infrastructure (5, 6). Economic accessibility, particularly the ability to afford healthcare, is another major factor influencing health. Research in China has highlighted that 17.95% of rice farmers in Hubei experience “financial hardship caused by medical expenses,” and 30.50% suffer from health poverty, indicating the severe economic constraints faced by farmers (7). Moreover, while the role of healthcare accessibility in improving health outcomes is recognized, most studies focus on individual dimensions without integrating them, thus overlooking how these dimensions interact and influence health outcomes across different demographic groups (8, 9). This gap in multidimensional research calls for a more comprehensive approach that considers geographic, economic, and social factors together to better understand and address farmers’ health vulnerabilities.

Healthcare accessibility is a multidimensional concept, encompassing geographic, economic, and social dimensions, all of which have significant implications for farmers’ health. This multidimensional understanding aligns with established theoretical frameworks in the literature, specifically Penchansky and Thomas’s (10) seminal model conceptualizing access through five dimensions—availability, accessibility, accommodation, affordability, and acceptability—with our study’s focus on geographic, economic, and social accessibility mapping onto this broader framework: geographic accessibility corresponds to Penchansky’s “accessibility” (spatial separation), economic accessibility to “affordability” (financial capacity), and social accessibility encompasses elements of “acceptability” (patient attitudes and cultural congruence) and “accommodation” (how services align with patient expectations). These dimensions are often studied separately, but in practice, they are deeply interrelated, and their impact on health outcomes is not always consistent. From a theoretical perspective, these dimensions are distinct but mutually reinforcing; however, empirical evidence often shows that their effects vary depending on the context and population being studied. Geographic accessibility, which refers to the physical distance and transportation barriers farmers’ face in accessing healthcare, has been widely acknowledged as a critical factor in health outcomes. In rural areas, long travel times, poor road conditions, and limited transportation options can delay diagnosis and treatment, leading to worse health outcomes (11). Economic accessibility, which addresses the financial ability of farmers to afford healthcare, is similarly significant. High out-of-pocket medical expenses, coupled with low health insurance coverage, frequently force farmers to delay or forgo treatment, which can lead to further health deterioration and perpetuate the cycle of poverty (7). Despite expanding health insurance coverage, the persistence of low reimbursement rates and complex claims processes often limits the effectiveness of insurance in rural areas, underscoring the critical role of financial accessibility in healthcare utilization.

Social accessibility, which refers to the social and cultural barriers that impact healthcare utilization, has received less attention in existing studies. However, its influence is undeniable. Farmers may be reluctant to seek care due to distrust of healthcare providers, lack of understanding of the healthcare system, or cultural beliefs that prioritize traditional medicine (12). Wu et al. (13) provide concrete evidence of such cultural influences, showing that among ethnic minority older adults in rural Southwestern China, healthcare-seeking behaviors are deeply embedded in traditional interpretations of illness, with self-care prioritized for minor ailments. This raises a critical question: if cultural orientations predispose rural residents toward self-care, what might shift their behavior toward formal healthcare utilization? Wei et al. (14), drawing on social capital theory, developed a dual-framework of trust comprising institutional trust (the willingness to engage with formal healthcare) and interpersonal trust (the choice between formal and informal systems); they demonstrated that when either form of trust is sufficiently established, culturally ingrained tendencies toward self-care can be overcome. Song et al. (15) provided empirical support for this theoretical insight. In their analysis of how rural doctors in China maintain long-term interpersonal trust with patients, they found that such trust is built through years of community integration, ensuring continuity of care even in challenging contexts such as palliative care.

However, interpersonal trust alone cannot compensate for systemic institutional weaknesses. Zhang (16) found that even with increased infrastructure investment, staff instability and weak community–provider interactions persistently constrain service uptake. This suggests that trust built with individual providers may erode when institutional support is lacking. The interplay between trust and institutional environments becomes particularly salient in policy implementation. Sun (17) examined Guizhou’s targeted medical assistance program and identified significant “cultural mismatches” between external medical teams and ethnic minority communities. These mismatches lead to resistance to Western medical diagnoses and non-adherence behaviors despite improved resource availability. This finding reveals a paradox: policy interventions aimed at improving access may fail precisely because they do not take into account crucial cultural and trust dynamics (14). The broader significance of these dynamics is confirmed by a national meta-analysis, which finds that health literacy, perceived service quality, and social trust now rival traditional determinants like income and insurance in shaping healthcare choices across China (18). Survey evidence from Jiangsu further specifies how trust operates. Patients value doctor communication yet remain skeptical of professional competence and treatment effectiveness, indicating that trust, cultivated through perceived quality and empathetic interaction, functions both as an outcome of positive experiences and as an independent driver of utilization (19). Finally, the cultural complexities of social accessibility are also reflected in family dynamics. Zhu and Ran (20) observed that among rural older adults with osteoporotic fractures, family support sometimes substitutes for formal care. This finding aligns with Wu et al.’s documentation of the prioritization of self-care, indicating that rural healthcare decision-making is embedded within a network of cultural expectations and family obligations, which may buffer against reliance on the formal system even when the latter is physically and financially accessible (14).

Although existing studies acknowledge the importance of these three dimensions, there is a significant gap in research that systematically compares their relative importance and explores their heterogeneous effects across different groups. Many studies focus on one dimension, such as geographic or economic accessibility, but fail to integrate them into a holistic framework that accounts for how these dimensions interact. This gap in the literature highlights the need for a multidimensional assessment of healthcare accessibility, which this study aims to address. By considering all three dimensions—geographic, economic, and social—this study seeks to provide a more comprehensive understanding of the factors that shape farmers’ health and to inform policies aimed at improving healthcare accessibility in rural areas.

To address these research gaps, this study constructs an integrated analytical framework of geographic, economic, and social accessibility to examine their respective and comparative impacts on farmers’ health. A series of robustness tests and instrumental variable (IV) approaches are used to assess the stability of the results. Furthermore, this study analyzes the heterogeneity of the effects across age, income, and educational groups. The contribution of this study is as follows: First, it proposes an innovative multidimensional framework that integrates geographic, economic, and social accessibility to comprehensively evaluate their collective impact on farmers’ health, thereby advancing beyond the typical single-dimensional approaches in existing literature. Second, it examines how healthcare resource accessibility affects farmers’ health across different points of the health distribution, emphasizing that individuals with different baseline health levels have distinct healthcare needs and benefit differently from improved accessibility. Third, it identifies heterogeneous effects of healthcare resources on farmers’ health across diverse demographic and socioeconomic groups, providing evidence for more targeted and equity-oriented health policies. The significance of this study lies in its potential to inform more effective and targeted policy interventions that address the multifaceted barriers farmers face in accessing healthcare, ultimately improving their health outcomes.

## 2 Variables and research methodology

### 2.1 Study population and study area

Data were obtained from the China Family Panel Studies (CFPS), a database that contains tracking data at the individual, household, and community levels, reflecting changes in Chinese society, economy, population, education, and health. The CFPS sample covers 31 provinces/autonomous regions, with a target sample size of 16,000 households. We identified farmers using two inclusion criteria: (1) household registration status (QA301), restricted to respondents who reported “agricultural household registration”; and (2) nature of employment (EGC2021), restricted to respondents who reported being “engaged in agricultural work (farming, forestry, animal husbandry, sideline production, and fishery)”. This study covers rural areas in 31 provinces/autonomous regions of China, including Beijing, Tianjin, and Shanghai. The study spans even-numbered years from 2012 through 2022, comprising six data periods.

### 2.2 Variable description

This study constructs a comprehensive research indicator system based on data from the China Family Panel Studies (CFPS) and the China Health and Family Planning Statistical Yearbook. The aim is to systematically analyze the impact of healthcare resource accessibility on farmers’ health. The key dependent variable in this study is health status, while the independent variables include geographic accessibility, economic accessibility, and social accessibility. Control variables include age, income, and educational attainment.

#### 2.2.1 Dependent variable

The dependent variable in this study is health status. It is based on the self-assessment question “How would you rate your health?” provided in the CFPS database. The ratings are scored on a 1–5 Likert scale, where 1 indicates “very healthy” and 5 indicates “very unhealthy.” For the purpose of analysis, we reverse the original scores so that higher scores represent better health. This self-reported health data reflects farmers’ subjective perceptions of their health, which helps supplement the limitations of objective health data.

#### 2.2.2 Independent variables

The key independent variables are the three dimensions of healthcare accessibility: geographic, economic, and social accessibility.

Geographic accessibility refers to the ease with which individuals can overcome spatial barriers to access healthcare services at the designated levels, based on their residential location. We measure this using data on provincial medical resource density and the mismatch between resources and demand, sourced from the CFPS and the China Health and Family Planning Statistical Yearbook. The medical resource density indicator is obtained by weighting the number of hospitals and primary healthcare institutions per province as reported in the China Health and Family Planning Statistical Yearbook. This indicator reflects the distribution density of healthcare resources, particularly in rural areas. The geographic accessibility index aims to capture the spatial convenience for farmers to access healthcare services. We constructed a composite index using two provincial-level variables from the China Health and Family Planning Statistical Yearbook: (1) health_density_c, the centered value of the weighted density of hospitals and primary healthcare institutions per province, reflecting the overall abundance of medical resources; and (2) facility_level, the level of healthcare facilities usually utilized by farmers (coded as 1 = village clinic, 2 = community health center, 3 = county hospital, 4 = tertiary hospital), representing the structural hierarchy of resource utilization. Both variables were standardized before analysis. To synthesize these two dimensions into a single measure, we employed principal component analysis (PCA), a dimensionality reduction technique that extracts orthogonal linear combinations of the original variables capturing the maximum variance. PCA helps identify underlying patterns and reduces multicollinearity, enabling us to construct a composite index that reflects the joint variation of resource density and facility level. Tables 1 and 2 present the PCA results and the loading matrix results.

**Table 1:** PCA results.

Principal component	Eigenvalue	Proportion of variance	Cumulative
Comp1	1.15357	0.5768	0.5768
Comp2	0.84643	0.4232	1.0000

**Table 2:** Loadings matrix.

Variable	Comp1	Comp2
Health_density_c	−0.7071	0.7071
Facility_level	0.7071	0.7071

The PCA loadings clarify the meaning of the two components. In Comp1, health_density_c and facility_level load with opposite signs (−0.7071 and +0.7071), indicating that this component reflects a mismatch between resource density and the level of care used. A higher Comp1 score therefore does not necessarily indicate better geographic access. By contrast, both variables load positively on Comp2 (0.7071 for each), suggesting that this component captures the joint improvement of resource availability and the level of care utilized. Although Comp2 explains slightly less variance than Comp1 (42.32% vs. 57.68%), it better matches the conceptual meaning of geographic accessibility. We therefore use the second principal component as the measure of geographic accessibility (GeoAccess), with higher values indicating better access.

Economic accessibility refers to farmers’ ability to afford healthcare services without jeopardizing their livelihood. It is measured by catastrophic health expenditures and the actual health insurance reimbursement rate. The data primarily comes from the CFPS, including the amount of medical expenses borne by individuals and the total hospitalization costs. The measure for catastrophic health expenditure is based on the definition of the World Health Organization and the World Bank, where catastrophic health expenditures are defined as situations where household medical out-of-pocket expenses exceed 25% of total household income, based on the “budget share method” (21). The actual health insurance reimbursement rate is measured by the ratio of hospitalization costs covered by health insurance to the total hospitalization expenses. The Economic Accessibility Index captures farmers’ ability to afford healthcare without financial distress. The index consists of two sub-indices derived from CFPS household expenditure and health insurance reimbursement information: (1) Catastrophic healthcare expenditure, a binary variable indicating whether out-of-pocket medical expenses exceed 25% of total household income; and (2) Actual health insurance reimbursement rate, calculated as the ratio of inpatient (hospitalization) costs reimbursed by health insurance to total inpatient (hospitalization) expenditure. Missing values in the economic variables were imputed using the median for each year. To ensure consistency (higher values indicate better economic accessibility), catastrophic healthcare expenditure was inversely encoded as 1 − catastrophic healthcare expenditure, where 1 represents no catastrophic expenditure. We then calculated the composite index EconAccess, an equally weighted average: EconAccess = 0.5 × (1 − catastrophic healthcare expenditure) + 0.5 × Actual health insurance reimbursement rate. This index ranges from 0 to 1, with higher values indicating better economic accessibility (no catastrophic expenditure and a higher reimbursement rate).

Social accessibility refers to farmers’ ability to access healthcare services from a social and cultural perspective, including their trust in healthcare providers, understanding of the healthcare system, and social support networks. Social accessibility captures the trust, satisfaction, and cultural acceptability of healthcare services from farmers’ perspectives. We constructed this index using two CFPS questions: qp602 (satisfaction with overall conditions of healthcare institutions) and qp603 (evaluation of the medical level at the facility used), both rated on a 1–5 Likert scale. We averaged these two items to obtain SocAccess, after excluding observations with invalid or missing responses. Missing values were imputed using year-specific medians. Higher values of SocAccess indicate greater satisfaction and trust, implying better social accessibility.

#### 2.2.3 Control variables

The control variables selected for this study are age, income, and educational attainment, as these are widely recognized in the literature as factors that influence health outcomes. Studies show that age is a key determinant of health, with older individuals typically facing more health problems, particularly in older populations (22). Income is closely linked to medical accessibility, with farmers with lower income bearing a higher healthcare burden and facing more limited healthcare coverage. In contrast, higher-income individuals generally have better access to quality healthcare services (23). Educational attainment, as a core indicator of socioeconomic status, is also significantly correlated with health outcomes. Individuals with higher educational levels generally have better health and a lower risk of mortality (24). Controlling for these variables effectively eliminates potential confounding factors, allowing for a more accurate assessment of the independent impact of healthcare resource accessibility on health outcomes. To ensure comparability, the education variable in CFPS was standardized by converting discrete educational categories into continuous years of education. The conversion follows the national educational system and CFPS’s official coding rules. For example, illiterate/semi-illiterate is equivalent to 0 years; primary school is 6 years; junior high school is 9 years; high school is 12 years; associate degree is set to 15 years (using the median); undergraduate is 16 years; master’s degree is 19 years; and doctoral degree is 22 years.

#### 2.2.4 Instrumental variables

In this study, instrumental variables (IVs) are used to address endogeneity: For geographic accessibility, we use the interaction term between “provincial hospital beds per thousand population” and “individual chronic disease prevalence” as an IV. This term is assumed to be exogenous because it reflects historical investments and macro-level policies and is not directly driven by unobservable individual health determinants. For economic accessibility, we use per capita social security and health expenditure relative to household non-medical consumption as an IV, capturing an exogenous adjusted healthcare price shock that affects accessibility but is not directly determined by an individual’s income. For social accessibility, we use “trust in doctors” as an IV. As a cultural and psychological factor, it affects healthcare utilization through perceived service quality and trust, but it is not assumed to directly determine health outcomes. The validity of these instruments was assessed based on relevance and exogeneity tests.

The variables used in this study, along with their definitions and descriptive statistics, are summarized in Table 3.

**Table 3:** Variable definitions and descriptive statistics.

Variable	Symbol	Description
Dependent variable		
Farmer health	HealthStatus	Self-reported health status of respondents in year t
Independent variables		
Geographic accessibility	GeoAccess	The geographic accessibility of medical services
Economic accessibility	EconAccess	The economic accessibility to healthcare services
Social accessibility	SocAccess	The social accessibility to healthcare services
Control variables		
Age	Age	Age of the respondent
Income	Income	Income of the respondent
Education	Education	Years of education

To ensure clarity in coefficient interpretation, Table 4 presents the coding direction for all key variables, indicating whether higher values represent better or worse health/accessibility.

**Table 4:** Variable coding directions.

Variable	Symbol	Interpretation of higher values
Dependent variable		
HealthStatus	HealthStatus Original scale: 1 = very healthy, 5 = very unhealthy; reverse-coded so that higher values indicate better health (1 = very unhealthy, 5 = very healthy).	Categorical variable, the higher value the better health
Independent variables		
Geographic accessibility	Derived from principal component analysis of health_density_c and facility_level	Higher values indicate better accessibility
Economic accessibility	Constructed as the equal-weighted average of negatively coded catastrophic health expenditure and the actual reimbursement rate	Higher values indicate better accessibility
Social accessibility	Social accessibility: Average of satisfaction with healthcare institution conditions (qp602) and medical level (qp603) (1 = very dissatisfied, 5 = very satisfied)	Higher values indicate better accessibility
Control variables		
Age	Respondent’s age in years	Continuous variable, higher values indicate older age
Income	Annual personal income	Continuous variable, higher values indicate higher income
Education	Years of education	Continuous variable, higher values indicate more years of education.

### 2.3 Research methodology

#### 2.3.1 Two-way fixed effects model

In this study, we use self-reported health status as the dependent variable, and geographic accessibility, economic accessibility, and social accessibility as the core independent variables. The control variables include age, income, and education level. Based on Grossman’s health capital theory, healthcare resource accessibility is considered a core element of health investment. To account for time-invariant unobserved heterogeneity at the individual level and common time trends affecting all farmers, we employ a two-way fixed effects model with individual fixed effects $\mu_{i}$ and year fixed effects $\lambda_{t}$ . The model is formulated as:


$$\begin{array}{l} {\text{HealthStatus}}_{\mathrm{it}}=\beta_{0}+\beta_{1}{\text{GeoAccess}}_{\mathrm{it}}+\beta_{2}{\text{EconAccess}}_{\mathrm{it}}\\ +\beta_{3}{\text{SocAccess}}_{\mathrm{it}}+\gamma_{1}{\mathrm{Age}}_{\mathrm{it}}+\gamma_{2}{\text{Income}}_{\mathrm{it}}\\ +\gamma_{3}{\text{Education}}_{\mathrm{it}}+\mu_{i}+\lambda_{t}+\varepsilon_{\mathrm{it}} \end{array}$$

where ${\text{HealthStatus}}_{\mathrm{it}}$ represents the health status of individual *i* in year t , ${\text{GeoAccess}}_{\mathrm{it}}$ , ${\text{EconAccess}}_{\mathrm{it}}$ and ${\text{SocAccess}}_{\mathrm{it}}$ represent geographic, economic, and social accessibility in year t , respectively; ${\mathrm{Age}}_{\mathrm{it}}$ , ${\text{Income}}_{\mathrm{it}}$ and ${\text{Education}}_{\mathrm{it}}$ are control variables; and $\varepsilon_{\mathrm{it}}$ is the error term; $\mu_{i}$ represents the individual fixed effect; $\lambda_{t}$ represents the year fixed effect.

#### 2.3.2 Quantile regression (QR) model

To analyze the heterogeneous effects of healthcare accessibility on health across different quantiles, we use the quantile regression model, which is specified as follows:


$$\begin{array}{l} {\text{HealthStatus}}_{\mathrm{it}}^{\tau }=\beta_{0}^{\tau }+\beta_{1}^{\tau }{\text{GeoAccess}}_{\mathrm{it}}+\beta_{2}^{\tau }{\text{EconAccess}}_{\mathrm{it}}\\ +\beta_{3}^{\tau }{\text{SocAccess}}_{\mathrm{it}}+\gamma_{1}^{\tau }{\mathrm{Age}}_{\mathrm{it}}+\gamma_{2}^{\tau }{\text{Income}}_{\mathrm{it}}\\ +\gamma_{3}^{\tau }{\text{Education}}_{\mathrm{it}}+\varepsilon_{\mathrm{it}}^{\tau } \end{array}$$

where ${\text{HealthStatus}}_{\mathrm{it}}^{\tau }$ represents the conditional health quantile at quantile τ ; ${\text{GeoAccess}}_{\mathrm{it}}$ , ${\text{EconAccess}}_{\mathrm{it}}$ and ${\text{SocAccess}}_{\mathrm{it}}$ are the explanatory variables; $\varepsilon_{\mathrm{it}}^{\tau }$ is the error term at quantile τ .

## 3 Empirical results

### 3.1 Association between healthcare accessibility and farmers’ health

Table 5 presents the results of the benchmark regression model. The table compares the coefficients obtained from the mixed OLS, two-way fixed effects, and random effects models. The Hausman test results reject the null hypothesis that “individual effects are uncorrelated with explanatory variables,” indicating that unobservable individual heterogeneity is systematically related to the core explanatory variables. Therefore, the two-way fixed effects model is used for the analysis.

**Table 5:** Benchmark regression results.

Model	Mixed OLS	Two-way fixed effects model	Random effects
Geographic accessibility	−0.006 (0.008)	−0.070*** (0.013)	−0.020*** (0.007)
Economic accessibility	1.190*** (0.034)	0.484*** (0.041)	0.940*** (0.031)
Social accessibility	0.069*** (0.010)	0.026** (0.010)	0.051*** (0.009)
Age	−0.023*** (0.000)	−0.021 (0.018)	−0.024*** (0.000)
Income	0.029** (0.013)	0.004 (0.016)	0.023* (0.012)
Education	0.012*** (0.004)	−0.000 (0.006)	0.007** (0.003)
Constant	1.169*** (0.140)	2.194*** (0.190)	1.550*** (0.129)
*F*-statistic	573.32	19.53	
*x* ^2^			5563.90
*R* ^2^	0.170	0.015	0.168
*N*	32,533	32,533	32,533
Individuals	18,241	18,241	18,241

***p < 0.01, **p < 0.05, *p < 0.1.

The results from the two-way fixed effects model show that the coefficient for geographic accessibility is −0.070 and statistically significant at the 1% level. This indicates an unexpected negative association: a one-unit increase in geographic accessibility is associated with a 0.070-unit decrease in self-rated health. The coefficient for economic accessibility is 0.484 and significant at the 1% level, confirming that improving financial access to healthcare significantly enhances farmers’ health. A one-unit increase in economic accessibility, such as higher reimbursement rates or reduced catastrophic health expenditure, is associated with a 0.484-unit improvement in self-rated health. This positive effect underscores the critical role of financial protection in facilitating healthcare utilization and improving health outcomes in rural areas. The coefficient for social accessibility is 0.026 and significant at the 5% level, indicating that higher levels of trust in healthcare providers and satisfaction with medical services are associated with modest but statistically significant improvements in farmers’ health. A one-unit increase in social accessibility corresponds to a 0.026-unit improvement in health status.

### 3.2 Quantile regression (QR)

Because farmers differ in their baseline health and vulnerability, those in poorer health rely more on timely, convenient, and affordable healthcare and thus derive greater health gains from improved accessibility, whereas healthier farmers exhibit diminishing marginal returns. To capture these heterogeneous impacts of accessibility across different health status groups, this study adopts the quantile regression methodology. Higher percentiles indicate better farmer health. Table 6 presents the results of the quantile regression analysis, showing the marginal effects of geographic, economic, and social accessibility at various health quantiles.

**Table 6:** Quantile regression results.

Quantile	10th percentile	25th percentile	50th percentile	75th percentile	90th percentile
Geographic accessibility	0.000 (0.000)	−0.021*** (0.007)	−0.021** (0.008)	0.011 (0.008)	0.054*** (0.014)
Economic accessibility	0.000 (0.000)	1.432*** (0.049)	1.837*** (0.056)	0.747*** (0.046)	1.089*** (0.116)
Social accessibility	0.000 (0.000)	0.031*** (0.009)	0.075*** (0.014)	0.081*** (0.011)	0.126*** (0.029)
Age	0.000 (0.000)	−0.030*** (0.001)	−0.017*** (0.002)	−0.022*** (0.000)	−0.020*** (0.001)
Income	0.000 (0.000)	0.053*** (0.012)	0.010 (0.013)	0.040*** (0.014)	−0.005 (0.024)
Education	0.000 (0.000)	0.027*** (0.002)	−0.007 (0.005)	−0.006 (0.004)	−0.008 (0.006)
Constant	1.000 (0.000)	0.203 (0.125)	1.256*** (0.165)	2.322*** (0.149)	3.363*** (0.282)
*N*	32,533	32,533	32,533	32,533	32,533
Individuals	18,241	18,241	18,241	18,241	18,241

***p < 0.01, **p < 0.05, *p < 0.1.

The quantile regression results reveal substantial heterogeneity in how healthcare accessibility affects farmers’ health across different points of the health distribution. At the 10th percentile—representing the poorest health status—all coefficients are zero. This occurs because the dependent variable (the reverse-coded self-rated health score, HealthStatus) has a lower bound of 1 (very unhealthy), and the 10th sample quantile exactly equals this minimum value. In our sample, the 10th quantile equals the lower bound of the outcome, and the estimated slope coefficients are zero, suggesting a strong floor effect at this quantile. Geographic accessibility exhibits a significant negative association at the 25th and 50th percentiles, indicating that better spatial access is associated with lower self-rated health among farmers with below-average or median health. The effect becomes insignificant at the 75th percentile but turns significantly positive at the 90th percentile (0.054, *p* < 0.01). Economic accessibility shows a consistently positive and highly significant effect from the 25th percentile upward. The coefficients are 1.432 (25th), 1.837 (50th), 0.747 (75th), and 1.089 (90th), all significant at the 1% level. This indicates that better economic access, reflecting lower financial barriers, is associated with substantially better health across almost the entire health distribution. The magnitude peaks at the median, implying that farmers with moderate health experience the greatest health gains from reduced economic constraints. Social accessibility also displays a positive gradient: coefficients increase from 0.031 (25th) to 0.126 (90th), all significant at the 1% level. This suggests that trust in healthcare providers and satisfaction with services matter more for farmers already in good health.

### 3.3 Instrumental variable (IV) regression

To further identify the causal relationship between healthcare accessibility and farmers’ health, we conduct the IV regression. Table 7 presents the results of the IV regression. The IVs used are the interaction of “provincial hospital beds per thousand population” with “individual chronic disease prevalence” for geographic accessibility, “per capita social security and health expenditure relative to household non-medical consumption” for economic accessibility, and “trust in doctors” for social accessibility.

**Table 7:** IV regression results.

Variable	IV regression
Geographic accessibility	−0.386** (0.156)
Economic accessibility	6.818*** (0.553)
Social accessibility	0.322** (0.127)
Age	−0.001 (0.003)
Income	0.047* (0.027)
Education	−0.015 (0.010)
Constant	−3.314*** (0.496)
*R* ^2^	−0.931
*N*	14,103
Individuals	9,058

***p < 0.01, **p < 0.05, *p < 0.1. Robust standard errors in parentheses.

The IV estimates indicate statistically significant effects of healthcare accessibility on farmers’ health. The coefficient for geographic accessibility is −0.386, significant at the 5% level. This indicates that, after controlling for endogeneity bias, a one-unit increase in geographic accessibility is associated with a 0.386-unit decrease in health scores. The coefficient for economic accessibility is 6.818, significant at the 1% level, suggesting that easing financial constraints yields substantial health returns. Given that EconAccess ranges from 0 to 1, the IV estimate (6.818) implies that a 0.1 increase in economic accessibility is associated with a 0.682-point increase in the health score (6.818 × 0.1), thereby playing a critical role in alleviating the health burden associated with catastrophic health expenditures. The coefficient for social accessibility is 0.322, significant at the 5% level, indicating that a one-unit increase in doctor-patient trust or satisfaction with healthcare services results in a 0.322-unit increase in health scores.

### 3.4 Heterogeneity analysis

This study conducts a heterogeneity analysis by grouping the sample based on age, income, education level, and region to examine how different groups respond to the accessibility of healthcare resources. Age, income, and educational level influence health status and medical needs, while regional disparities reflect differences in economic development, healthcare infrastructure, and policy environments. By conducting a subgroup analysis, we can reveal the heterogeneity in healthcare accessibility among different groups and provide more targeted policy recommendations. Table 8 shows the results of the heterogeneity analysis by age, income, and education level.

**Table 8:** Heterogeneity analysis.

Variable	Age			Income			Education		
	Youth	Middle-aged	Older	Low-income	Middle-income	High-income	Low-education	Middle-education	Higher-education
Geographic accessibility	−0.033 (0.026)	−0.096*** (0.023)	−0.093*** (0.024)	−0.003 (0.113)	−0.085*** (0.017)	0.037 (0.056)	−0.081*** (0.015)	−0.017 (0.034)	−0.022 (0.122)
Economic accessibility	0.402*** (0.112)	0.482*** (0.070)	0.519*** (0.063)	0.221 (0.462)	0.485*** (0.051)	0.543** (0.223)	0.502*** (0.044)	0.271** (0.132)	0.804 (0.708)
Social accessibility	0.066*** (0.025)	0.000 (0.018)	0.036** (0.018)	0.117 (0.099)	0.022* (0.013)	0.110** (0.050)	0.023** (0.012)	0.027 (0.029)	−0.173 (0.142)
Age	−0.046 (0.044)	−0.027 (0.049)	−0.030 (0.027)	0.139 (0.354)	−0.031 (0.025)	−0.021 (0.022)	0.003 (0.026)	−0.045 (0.043)	−0.406 (0.290)
Income	0.012 (0.028)	0.018 (0.029)	−0.027 (0.035)	0.034 (0.140)	0.092 (0.083)	0.093 (0.076)	0.009 (0.019)	−0.031 (0.041)	0.066 (0.146)
Education	0.013 (0.015)	−0.018 (0.014)	0.002 (0.009)	−0.076 (0.059)	0.003 (0.007)	0.040 (0.052)	0.000 (—)	−0.005 (0.034)	0.008 (0.317)
Constant	1.602 (0.981)	2.162*** (0.321)	2.615*** (0.591)	2.338** (1.063)	1.293 (0.845)	0.791 (0.934)	2.034*** (0.226)	2.744*** (0.652)	−4.971 (6.767)
*N*	3,860	8,889	8,455	204	16,115	1,102	19,861	2,401	131
Individuals	1,641	3,593	3,343	98	6,867	511	7,731	979	59
Adjusted *R*^2^	0.451	0.449	0.441	0.510	0.476	0.513	0.466	0.573	0.467

***p < 0.01, **p < 0.05, *p < 0.1.

First, the study divides age into three groups: (1) Youth (18–35 years), (2) Middle-aged (36–55 years), and (3) Older (56 years and above). Younger farmers are generally healthier and at the peak of physical functioning. Most individuals in this age range do not suffer from serious chronic diseases and are less likely to be affected by diseases caused by lifestyle factors. As individuals reach middle age (36–55 years), health problems begin to emerge, influenced by work pressure, lifestyle changes, and the onset of some chronic diseases. In the older group (56 years and above), health conditions generally decline, with an increased risk of chronic diseases and age-related conditions. Public health studies indicate that individuals over the age of 56 face significant health challenges.

Second, income is categorized into three groups: (1) Low income (<10,000 RMB), (2) Middle income (10,000–30,000 RMB), and (3) High income (>30,000 RMB). Farmers with lower income generally face poorer living conditions and allocate a higher proportion of their income to medical expenses, which contributes to increased health risks. According to existing socio-economic studies, farmers with an income of less than 10,000 RMB usually face significant health burdens and limited access to medical security. In contrast, middle-income groups have a lower healthcare burden but may still face issues such as insufficient health security. Middle-income groups’ health outcomes are more influenced by economic protection. High-income individuals can typically afford high-quality medical services and health management, leading to relatively better health.

Third, the study divides the sample based on education into: (1) Lower educational attainment (Primary school and below, 0–6 years), (2) Middle level of educational attainment (Junior high school to high school, 7–12 years), and (3) Higher educational attainment (Associate degree and above, 13+ years). This grouping aims to test how education level, which represents the ability to obtain and understand health information, moderates the efficiency of health transformation through healthcare accessibility. Farmers with lower educational attainment are largely influenced by traditional experiences and community information, and they may have limited health literacy as well as challenges in understanding and trusting modern medical knowledge. They may view healthcare institutions as places to address only major health crises rather than as routine venues for preventive care and chronic disease management. Medium-education farmers, representing the main body of rural society, have basic health information comprehension and a higher level of rational decision-making, with a fundamental understanding and trust in the modern medical system. They are the core group covered by the New Rural Cooperative Medical Scheme. This group has normalized their healthcare utilization but may face limitations in the equity and efficiency of resource allocation. The high-education group represents rural residents with higher health human capital, possessing strong information retrieval skills, scientific health concepts, and proactive health investment awareness. They are not only more efficient in utilizing accessible healthcare resources but are also more likely to seek high-quality services, overcoming some geographical and economic barriers.

Fourth, the study conducts a regional heterogeneity analysis by grouping the sample into four major regions of China: Eastern, Central, Western, and Northeastern. This classification follows the standard regional division used in Chinese official statistics, reflecting disparities in economic development, healthcare infrastructure, and policy priorities. The Eastern region comprises economically developed coastal provinces and municipalities (Beijing, Tianjin, Hebei, Shanghai, Jiangsu, Zhejiang, Fujian, Shandong, Guangdong, Hainan). The Central region includes inland provinces with moderate development levels (Shanxi, Anhui, Jiangxi, Henan, Hubei, Hunan). The Western region covers less developed western provinces and autonomous regions (Inner Mongolia, Guangxi, Chongqing, Sichuan, Guizhou, Yunnan, Tibet, Shaanxi, Gansu, Qinghai, Ningxia, Xinjiang). The Northeastern region consists of the three traditional industrial provinces (Liaoning, Jilin, Heilongjiang). By examining regional differences, we can uncover how healthcare accessibility varies across areas with distinct economic and institutional contexts, providing insights for targeted regional health policies. Due to space constraints in the main tables, the regional heterogeneity analysis is presented separately in Table 9.

**Table 9:** Heterogeneity analysis.

Variable	Region			
	Eastern	Central	Western	Northeastern
Geographic accessibility	−0.063** (0.031)	−0.057** (0.029)	−0.082*** (0.019)	−0.070* (0.041)
Economic accessibility	0.329*** (0.092)	0.659*** (0.088)	0.513*** (0.061)	0.286** (0.119)
Social accessibility	−0.006 (0.022)	−0.001 (0.023)	0.052*** (0.017)	0.018 (0.029)
Age	0.067 (0.086)	0.023 (0.042)	−0.034* (0.019)	−0.184 (0.114)
Income	−0.018 (0.035)	0.061* (0.032)	0.003 (0.026)	−0.077 (0.051)
Education	0.000 (0.021)	−0.002 (0.011)	−0.001 (0.008)	0.013 (0.022)
Constant	2.426*** (0.503)	1.549*** (0.367)	2.074*** (0.282)	3.825*** (0.794)
*N*	4,838	5,673	10,724	2042
Individuals	1916	2,259	4,116	792
Adjusted *R*^2^	0.514	0.499	0.465	0.541

***p < 0.01, **p < 0.05, *p < 0.1.

According to Table 8, in terms of age, geographic accessibility shows significant negative effects among the middle-aged and older groups, with coefficients of −0.096 and −0.093, respectively, both larger in magnitude than the −0.033 observed in the youth group. Economic accessibility exhibits a positive and increasing trend across age groups, with coefficients of 0.402 (youth), 0.482 (middle-aged), and 0.519 (older), all significant at the 1% level, suggesting that older farmers benefit more from enhanced economic access to healthcare. Social accessibility is significantly positive for the youth group and the older group, but not significant for the middle-aged group, implying that younger and older farmers may place greater value on relational and trust-based aspects of care. In the income dimension, geographic accessibility is significantly negative only for the middle-income group, indicating a negative association between geographic accessibility and self-rated health in this group. Economic accessibility is significantly positive across all income groups, with coefficients of 0.485 (middle-income), 0.543 (high-income), and a non-significant 0.221 for the low-income group. This suggests that middle- and high-income farmers are more responsive to reductions in financial barriers. Social accessibility is significantly positive for the middle-income and high-income groups, but not for the low-income group. The strong positive effect among high-income farmers suggests that as income increases, healthcare expectations shift toward service quality and trust. In terms of education, geographic accessibility is significantly negative only for farmers with lower educational attainment, indicating a negative association between geographic accessibility and self-rated health in this subgroup. Economic accessibility is significantly positive for both farmers with lower educational attainment and the middle-education group, with the largest effect observed among the former. Social accessibility is significantly positive only for farmers with lower educational attainment, suggesting that they rely more on interpersonal trust and subjective evaluations of care. In contrast, no significant effects are found for geographic or social accessibility among middle- and high-education groups, possibly due to greater health literacy and more proactive healthcare-seeking behavior.

According to Table 9, in terms of regional heterogeneity, the effects of healthcare accessibility on farmers’ health vary substantially across China’s four major economic zones. Geographic accessibility exhibits significant negative effects in all regions, with the largest coefficient observed in the Western region (coefficient = −0.082, significant at the 1% level), followed by the Northeastern region (−0.070, significant at the 10% level), the Eastern region (−0.063, significant at the 5% level), and the Central region (−0.057, significant at the 5% level). Economic accessibility shows significant positive effects across all regions, with the largest coefficient in the Central region (0.659, significant at the 1% level), followed by the Western region (0.513, significant at the 1% level), the Eastern region (0.329, significant at the 1% level), and the Northeastern region (0.286, significant at the 5% level). This indicates that reducing financial barriers to healthcare is particularly impactful in the Central and Western regions, where farmers may face higher economic vulnerability. Social accessibility is significantly positive only in the Western region (coefficient = 0.052, significant at the 1% level), and not significant in the other three regions, implying that trust-based and relational aspects of healthcare play a more critical role in the West, possibly due to stronger community ties or greater reliance on local healthcare providers.

## 4 Conclusion and implications

In the context of global agricultural systems and food security, improving farmers’ health capital is crucial. However, farmers face multiple health risks, particularly in rural areas where accessibility to healthcare resources directly influences health outcomes. Drawing on data from the China Family Panel Studies (CFPS), this study employs the two-way fixed effects model, quantile regression, and instrumental-variable (IV) regression to examine the impact of geographic, economic, and social accessibility on farmers’ health. A series of robustness tests confirms the reliability of the results, and the study further explores heterogeneity across different groups.

The benchmark results from the two-way fixed effects model indicate that economic accessibility has the most significant impact on farmers’ health, with its coefficient far exceeding those of geographic and social accessibility. This suggests that reducing the financial burden of medical expenses and improving the healthcare security system is the most direct driver of health improvements for the rural population. The significantly negative coefficient for geographic accessibility presents a counterintuitive finding. This phenomenon may stem from a selection bias within the sample, where individuals with poorer health are forced to travel long distances to seek higher quality medical resources, thus manifesting in cross-sectional data as an illusion that those in worse health have better geographic accessibility. This pattern points to possible reverse causality. The positive effect of social accessibility, while modest, is robust, indicating that doctor-patient trust and service satisfaction act as facilitators of health improvement, although their full effect may depend on the fulfillment of other basic conditions. Quantile regression further reveals significant heterogeneity in the effects of healthcare accessibility across different health levels. Economic accessibility maintains a significantly positive impact across all quantiles, but its effect exhibits an inverted U-shape, with higher values in the middle and lower values at the ends. This indicates that the marginal health benefits from reducing economic constraints are greatest for farmers at moderate health levels. This group may be at a critical threshold of health decline, where timely access to medical services can effectively interrupt the progression of illness. The effect of social accessibility increases with higher quantiles, suggesting that higher-order needs, such as trust and service quality, become more prominent among groups with better health status. This reflects a hierarchy of health needs. Once basic survival-type medical needs are satisfied, farmers’ expectations of healthcare services shift toward experiential demands. The trajectory of geographic accessibility, showing zero at the lowest quantile, negative at middle quantiles, and turning positive at the highest quantile, further corroborates the suspicion of reverse causality from the benchmark results. At the lowest quantile, the estimated slope coefficients are zero, consistent with a strong floor effect because the outcome is bounded below. In contrast, those with excellent health, represented by the highest quantile, can leverage geographic advantages for preventive care. Instrumental variable estimation results, after correcting for endogeneity bias, show a substantial increase in the coefficients for all dimensions, suggesting that the IV estimates are consistent with the main findings. The coefficient for economic accessibility expands dramatically to 6.818, indicating that the benchmark results may have significantly underestimated the true health effect of financial protection due to omitted variables or measurement error. The negative coefficient for geographic accessibility further amplifies in the IV estimation, statistically substantiating the presence of reverse causality. This suggests that the observed negative association is driven by health-related care-seeking behavior (reverse causality), rather than a detrimental effect of geographic accessibility on health. The coefficient for social accessibility also increases, suggesting that after removing confounding factors, the independent contribution of social trust to health becomes more pronounced. From an age perspective, the negative effect of geographic accessibility is stronger for middle-aged and older groups, reflecting their reality of seeking medical care across regions after falling ill. The increasing effect of economic accessibility with age highlights the older population’s higher dependence on financial protection for healthcare. From an income perspective, the significant effect of economic accessibility on middle and high income groups, but not on the low-income group, might seem paradoxical. This could be explained by differences in effective demand. Although the low income group faces the tightest financial constraints, their healthcare decisions are simultaneously influenced by multiple factors like information access and health awareness. Simply improving economic accessibility without complementary support may not fully translate into healthcare utilization. From an education perspective, geographic accessibility is significantly negative while social accessibility is significantly positive for farmers with lower educational attainment, suggesting that the geographic pattern may reflect care-seeking behavior related to reverse causality, whereas trust-related factors are positively associated with health. Regional heterogeneity analysis further demonstrates substantial variation across economic zones. Geographic accessibility exhibits significant negative effects in all regions, with the largest magnitude observed in the Western region, followed by the Northeastern, Eastern, and Central regions. This consistent negative pattern across all areas reinforces the counterintuitive finding that individuals with poorer health tend to travel farther for care, with this phenomenon being most pronounced in the underdeveloped West. Economic accessibility shows significant positive effects across all regions, with the largest coefficients in the Central and Western regions. This indicates that reducing financial barriers to healthcare is particularly impactful in areas where farmers face higher economic vulnerability, confirming that financial protection mechanisms are most critical in less developed economies. Social accessibility is significantly positive only in the Western region and not significant in the other three regions. This unique finding implies that trust-based and relational aspects of healthcare play a more critical role in the West, possibly due to stronger community ties, greater reliance on local healthcare providers, or the relative weakness of formal institutional arrangements in this region.

The main strength of this study lies in its integrated framework that jointly considers geographic, economic, and social accessibility, and examines their effects on farmers’ health across the health distribution and across demographic and socioeconomic subgroups. However, this study has several limitations. First, in terms of health status measurement, our reliance on self-reported data, while common in population surveys, may be subject to social desirability bias and cross-cultural variations in health perception, and the lack of objective indicators like clinical diagnoses could introduce subjective bias. Second, the use of province-level geographic accessibility measures is insufficient, as these aggregate indicators mask critical spatial barriers such as actual travel distance, transportation infrastructure, and intra-provincial disparities, thereby potentially overlooking localized inequities in healthcare access. Third, our measurement of socio-cultural dimensions is narrow, relying on two subjective indicators that fail to capture broader factors like traditional health beliefs, information access, or community support, which may lead to an underestimation of their impact on healthcare-seeking behavior and health outcomes. Fourth, our static panel approach captures only average effects over the sample period, failing to reveal how the impact of healthcare accessibility on health evolves over time, particularly in response to major policy shifts that may produce different short- versus long-term effects. Based on the results, policymakers should prioritize economic accessibility, which exerts the largest health effect, particularly for older farmers and those in Central and Western regions. Improving local primary care infrastructure may reduce long-distance care-seeking, thereby weakening reverse-causality concerns in the geographic accessibility measure. Fostering patient-provider trust offers additional health gains, especially in the Western region. Tailored approaches are essential. For farmers with lower educational attainment, strengthening local primary care and community-based services may reduce long-distance care-seeking, which helps mitigate potential reverse-causality concerns in the geographic accessibility measure, while enhancing social accessibility, including trust and service experience, can provide additional health benefits. Similarly, farmers with lower income require a combination of health education and financial protection, whereas higher-income farmers place greater value on improved service quality. A multidimensional strategy integrating financial protection, infrastructure investment, and community-embedded care is necessary to address heterogeneous barriers and improve health outcomes across rural China.

## Data Availability

The raw data supporting the conclusions of this article will be made available by the authors, without undue reservation.
